# Green Synthesis of Trifluoromethanesulfonyl Fluoride as an Eco-Friendly Alternative to SF_6_ Gas Insulation and Analysis of Its Acute Inhalation Toxicity

**DOI:** 10.3390/molecules30102241

**Published:** 2025-05-21

**Authors:** Shile Wang, Li Dong, Ruichao Peng, Hongding Tang

**Affiliations:** 1 College of Chemistry & Molecular Sciences, Wuhan University, Wuhan 430072, China; shile_wang@whu.edu.cn; 2 Quanzhou Yuji Advanced Materials Co., Ltd., Quanzhou 362804, China; dongli@yujigroup.com

**Keywords:** TFSF, green synthesis, halogen exchange, acute inhalation toxicity, SF_6_ alternative

## Abstract

This study demonstrates an eco-friendly synthesis of trifluoromethanesulfonyl fluoride (TFSF) as a sustainable SF_6_ alternative. Optimized halogen exchange reactions using CF_3_SO_2_Cl/KF (3:1 ratio) with crown ether catalysis at low temperatures achieved 65% TFSF yield (97.9% purity). Scale-up trials in pressurized reactors showed >50% conversion and >90% selectivity. Acute inhalation tests (OECD standards) on Sprague-Dawley rats revealed transient toxicity at 20,000 ppm (4 h exposure), with survival rates >66% and LC_50_ exceeding 22,600 ppm—significantly safer than SF_6_. These findings confirm TFSF’s technical viability and low toxicity, positioning it as a practical insulating medium to curb SF_6_ emissions. The methodology highlights precision halogen exchange control and systematic safety validation, offering actionable solutions for industrial adoption.

## 1. Introduction

The global movement towards carbon neutrality has resulted in two key pressures for the power sector: the curtailment of greenhouse gases, with a particular focus on non-CO_2_ emissions. Following the establishment of the 2021 Glasgow Climate Pact, regulatory measures have been implemented with a specific focus on sulfur hexafluoride (SF_6_) [1]. This compound, a prevalent electrical insulator, possesses a global warming potential (GWP) of 23,500 [2], as well as the capacity to induce severe climate impacts and demonstrate millennia-long atmospheric persistence. This compound’s environmental footprint proves especially problematic in high-consumption regions where SF_6_ leakage now poses critical operational and ecological risks [3,4,5,6], driving urgent demand for sustainable dielectric alternatives.

Trifluoromethanesulfonyl fluoride (TFSF) is a chemically versatile compound that is notable for its compact structure as a perfluoroalkyl sulfonyl halide. This characteristic has attracted scientific interest, leading to research on its hybrid functionality. The substance’s low boiling point (−25 °C) [7] and moderate vaporization energy (23.4 kJ/mol) [8] facilitate gas-phase handling at ambient temperatures with pressure-controlled liquefaction. From a structural perspective, TFSF combines a lipophilic trifluoromethyl group, which is valued in medicinal chemistry for its ability to enhance membrane permeability and metabolic stability [9], with a sulfonyl fluoride unit. This combination demonstrates unparalleled resistance to redox degradation. In contrast to conventional sulfonyl halides, this configuration exhibits reduced reactivity towards nucleophiles and unsaturated bonds while preserving thermal robustness. The reactivity exhibited by TFSF is predominantly attributable to its sulfur center, thereby manifesting environment-dependent transformations [10]. It has been demonstrated that alkaline conditions are conducive to the rapid hydrolysis of sulfonate salts [11]. Conversely, neutral or mildly acidic media have been shown to preserve stability. The compound has been demonstrated to react selectively with alcohols and amines under controlled conditions, thus enabling precise functionalization. This characteristic is of significant value in the synthesis of fluorinated surfactants and covalent drug conjugates [12,13]. In view of the high vapor pressure of TFSF and its susceptibility to hydrolysis under alkaline conditions, it is imperative that it is stored as a liquid at low temperatures in sealed, moisture-free stainless steel cylinders.

Despite its apparent potential, large-scale production of fluorinated sulfur species such as TFSF remains constrained by legacy synthetic methods that rely on elemental fluorine or anhydrous hydrogen fluoride [14,15,16,17]. These conventional approaches have been shown to introduce significant safety hazards and face limitations in terms of selectivity and scalability [18,19,20]. Consequently, they are incompatible with modern standards for green manufacturing. In response to these challenges, the present study proposes an environmentally conscious halogen exchange synthesis conducted in a continuous-flow reactor system. This method is notable for its exclusion of fluorine gas and other hazardous reagents, thus ensuring enhanced safety, process consistency, and industrial feasibility.

In the context of power infrastructure, the integration of novel dielectric gases necessitates the adherence to stringent safety criteria, extending beyond the scope of synthesis. As a volatile compound with potential inhalation exposure pathways, TFSF requires thorough toxicological assessment prior to deployment [21]. Acute inhalation toxicity testing is central to regulatory evaluation, particularly under the framework of OECD Test Guideline 403 [22], which defines the parameters for occupational exposure limits and emergency response planning. In contrast to the paucity of data concerning many perfluorinated compounds, TFSF currently lacks publicly available inhalation safety profiles, an absence that may hinder both risk assessment and regulatory approval.

In order to address this gap in the literature, this study presents the first inhalation toxicity evaluation of TFSF. This evaluation was conducted in accordance with OECD standards. A 4 h exposure at 20,000 ppm (the threshold concentration that defines Category 5, the lowest acute toxicity classification) was selected for the purpose of assessing potential health risks and workplace compatibility. This evaluation provides essential toxicological context to complement the synthetic advances, establishing a comprehensive foundation for the safe and sustainable replacement of SF_6_ in high-voltage applications, in alignment with global carbon-neutrality goals.

## 2. Green Synthesis of TFSF

### 2.1. Chemical Background and Rationale

TFSF, the smallest perfluoroalkyl sulfonyl halide, boils at −25 °C and exists as a compressible gas with a faint odor. The trifluoromethyl group enhances lipophilicity and stability, while the sulfonyl fluoride moiety ensures thermal and redox resistance. The aforementioned dual functionality facilitates applications in battery electrolytes, insulating gases, and precision fluorination.

### 2.2. Synthesis Route and Experimental Setup

Utilizing TFSF’s verified dielectric characteristics as a foundation, a liquid-phase halogen exchange synthesis was engineered through the implementation of CF_3_SO_2_Cl and KF in conjunction with crown ether phase-transfer catalysis. The 3:1 KF stoichiometry that has been optimized has established the industrial viability of this route for scalable SF_6_-alternative production, as outlined in Equation (1).
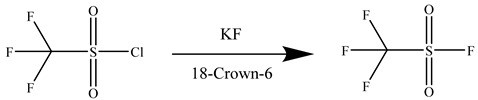
(1)

**Figure 1:**
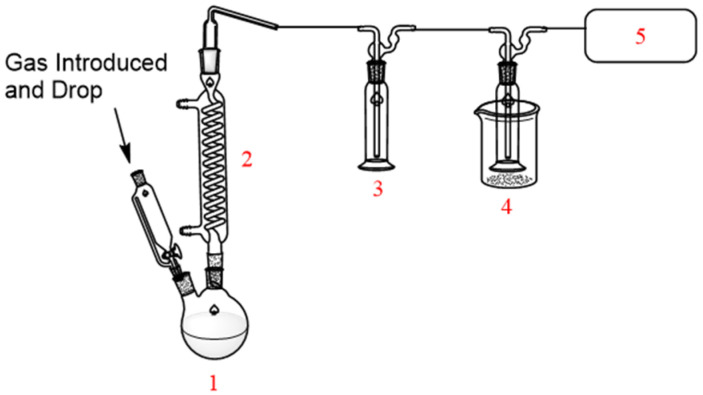
Schematic of the TFSF synthesis apparatus. (1) Reaction flask; (2) coil condenser; (3–4) cold traps; (5) gas collection bag.

Figure 1 shows a schematic of the experimental setup, which includes the reaction flask, a coil condenser, dual cold traps, and a gas collection bag.

### 2.3. Product Characterization

**Figure 2:**
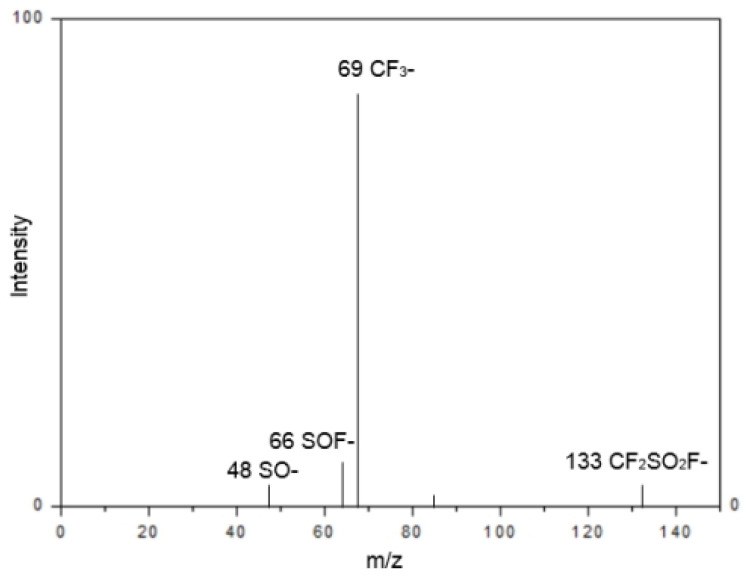
Representative GC-MS spectrum of the crude TFSF product.

Gas chromatography–mass spectrometry (GC-MS) analysis confirmed the synthesis of TFSF, revealing diagnostic fragmentation patterns at m/z 48 (M–SO), 66 (M–SOF), 69 (M–CF_3_), and 133 (M–CF_2_SO_2_F). The presence of these characteristic peaks serves to validate the molecular structure, thereby demonstrating the efficacy of the halogen exchange process. This exchange is achieved through targeted bond cleavages within the sulfonyl fluoride framework (Figure 2).

### 2.4. Process Optimization

#### 2.4.1. Feedstock Ratio Optimization

It was demonstrated that varying the KF:CF_3_SO_2_Cl ratio had a marked effect on reaction performance. The yield increased from 48% at a ratio of 1:1 to a peak of 65% at a ratio of 3:1, accompanied by a substantial improvement in purity (from 90.0% to 97.9%). It was demonstrated that additional increases to the ratio of 4:1 and 5:1 did not result in substantial gains in yield. This finding indicates that a saturation point has been attained. Consequently, the 3:1 molar ratio is identified as optimal, achieving a balance between reagent efficiency and product quality without an undesirable excess of fluoride reagent (Table 1).

#### 2.4.2. Reaction Temperature Influence

The data demonstrate that within the temperature range of −10 °C to 0 °C, there is a substantial enhancement in both yield (from 21% to 65%) and purity (from 96.2% to 97.9%) with increasing temperature. However, further temperature elevation above 0 °C has been shown to result in significant declines in both metrics. Notwithstanding the suppression of side reactions at −10 °C, the overall productivity is constrained by low reactivity. Conversely, 0 °C offers an optimal balance between kinetic efficiency and thermal control, thereby achieving the highest yield and purity. Consequently, 0 °C is identified as the optimal temperature for the synthesis of TFSF within this system (Table 2).

### 2.5. Scale-Up and Batch Synthesis

**Figure 3:**
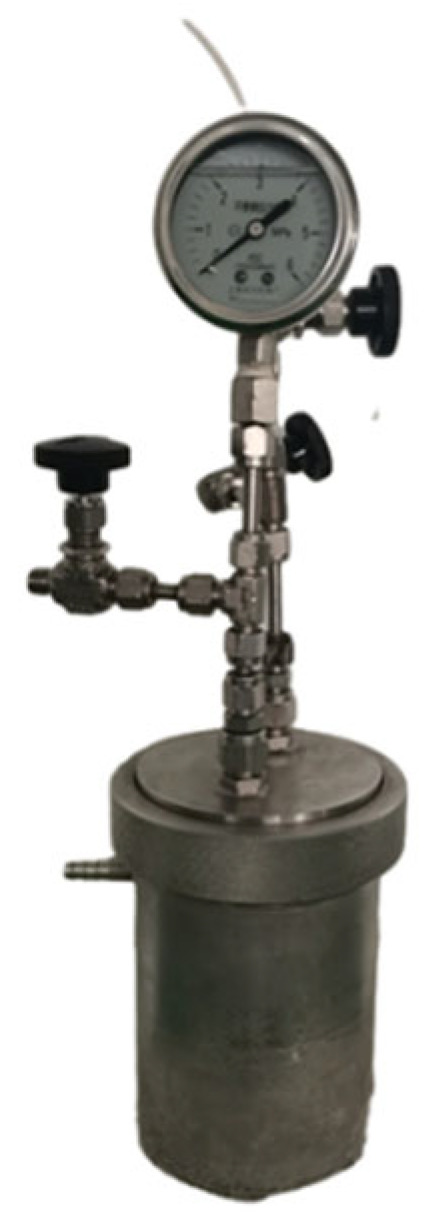
High-pressure reactor setup for batch synthesis of TFSF.

The scale-up optimization process in a 500 mL pressurized reactor has been shown to achieve industrial-grade TFSF synthesis through the balancing of thermal and kinetic factors. The process is characterized by the maintenance of a temperature of 50 °C, with controlled KF feeding and stabilized pressure at 0.5 MPa. This is achieved through the suppression of byproduct formation, while enabling efficient TFSF isolation through fractional distillation. The protocol exhibits 94% selectivity at 58% conversion, metrics that substantiate scalability for continuous-flow systems. It is imperative to note that the operational window is instrumental in preventing thermal degradation of CF_3_SO_2_Cl, thereby ensuring a product purity level that exceeds 97%, which is comparable to the results obtained from bench-scale experiments (Figure 3 and Table 3).

The results from the kilogram-scale experiments satisfied the predefined performance criteria, demonstrating an average feedstock conversion rate exceeding 50% and target product selectivity above 90%.

### 2.6. Summary

This research details an eco-conscious TFSF synthesis via precision-controlled halogen exchange, achieving 97.9% purity through optimized stoichiometry and cryogenic conditions. High-pressure reactor trials demonstrated scalable production with 94% selectivity, validating industrial viability while advancing sustainable alternatives to SF_6_ in energy infrastructure.

## 3. Acute Inhalation Toxicity Study of TFSF

### 3.1. Test System

#### 3.1.1. Experimental Animals

Sprague-Dawley (SD) rats, certified as specific pathogen-free (SPF), were chosen for this acute inhalation toxicity study. A total of six animals (three male and three female) were procured from a qualified supplier in Beijing (Production License SCXK (jing)2024-0001). The animals were selected for their well-characterized genetic background, reduced inter-individual variability, and ease of handling (Table 4).

Individual animals were uniquely identified using tail markings, and cage cards were used to record pertinent experimental details including animal IDs, group assignments, and test dates.

#### 3.1.2. Housing and Maintenance

Animals were housed in barrier facilities with stainless steel racks and plastic cages, with 2–3 animals per cage. The environment was maintained at 20–26 °C with a relative humidity of 40–70% and a 12 h light/dark cycle. Sterilized feed (obtained from a certified supplier) and filtered, autoclaved municipal water were provided ad libitum. Bedding was replaced weekly, and all procedures followed established Standard Operating Procedures approved by the Institutional Animal Care and Use Committee.

### 3.2. Experimental Methods

#### 3.2.1. Standards and Guidelines

This study was performed in accordance with OECD Test Guideline 403 [22] for acute inhalation toxicity testing of chemicals. The test protocol was designed to evaluate the acute toxicity profile of TFSF via inhalation.

#### 3.2.2. Test Substance Preparation and Exposure

**Figure 4:**
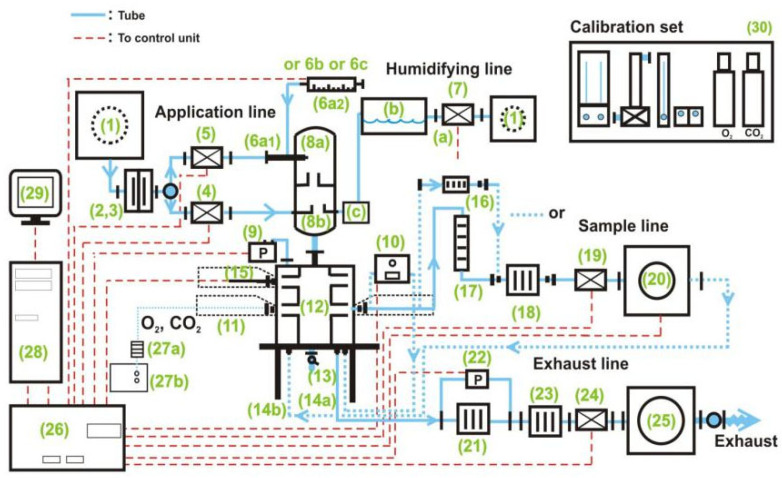
Schematic diagram of the nose-only inhalation exposure system (1, air supply system and pressure reducing system; 2–3, filter system; 4–5, mass flow controllers (air in); 6, aerosol generation; 7, humidifying line; 8, aerosol conditioning; 9, pressure sensor; 10, concentration measuring unit; 11, animal cage; 12–14, head–nose-only unit; 15, Tem/Hum sensor; 16–20, sample line; 21–25, exhaust line; 27, O_2_/CO_2_ measuring unit; 26, 28, 29, control and regulation system; 30, calibration set).

TFSF vapor was blended with purified air from liquefied cylinders and delivered via dynamic inhalation systems to a nose-only exposure chamber. Precision aerosol controllers maintained consistent flow rates (1.2 L/min) and uniform gas distribution, achieving ≤5% concentration variance during 4 h rodent toxicity assessments under OECD Test Guideline 403 [22] (Figure 4).

#### 3.2.3. Dosage Design and Grouping

The selected exposure concentration (20,000 ppm for 4 h) was based on the threshold defined in OECD Test Guideline 403 [22], where substances with LC_50_ values exceeding 20,000 ppm under a 4 h exposure are classified as having the lowest acute toxicity (Category 5). A test group consisting of six Sprague-Dawley rats (three males and three females) was exposed to a nominal TFSF concentration of 20,000 ppm for a period of four hours.

#### 3.2.4. Exposure and Monitoring

Researchers housed SD rats in nose-only restrainers during 4 h exposures, maintaining TFSF flow at 230 mL/min with 10 L/min air dilution. Integrated sensors tracked ambient conditions (temperature, humidity, O_2_/CO_2_) every 10 min, while 2,2,2-trifluoroethanol traps collected periodic aerosol samples for GC validation of exposure consistency.

#### 3.2.5. Post-Exposure Observation and Data Collection

Post-exposure, animals underwent 14-day recovery with clinical monitoring for neurobehavioral symptoms (tremors, lethargy), respiratory function, and weight trends. Terminal CO_2_ euthanasia preceded macroscopic organ assessments, revealing no exposure-linked pathologies. Critical endpoints were tracked at 30 min, 24 h, and weekly intervals.

### 3.3. Data Analysis and Results

#### 3.3.1. Exposure Environment

GC analysis verified TFSF exposure levels at 22,609.9 ± 735.4 ppm, aligning with target concentrations. Ambient conditions remained stable at 22.82 ± 0.79 °C and 47.56 ± 1.95% humidity, with oxygen (20.12 ± 0.05%) and carbon dioxide (0.07 ± 0.02%) levels complying with OECD Test Guideline 403 [22], ensuring controlled experimental validity.

#### 3.3.2. Mortality and Clinical Observations

Two fatalities (one male, one female) occurred within 24 h post-20,000 ppm exposure. Surviving subjects exhibited transient neurobehavioral abnormalities (lethargy, tremors) resolving by Day 7, with body weights recovering to baseline. Gross necropsy identified no exposure-related pathologies (Table 5).

#### 3.3.3. Body Weight Changes

Surviving rats exhibited reversible weight fluctuations, with 7–9% mass loss during Days 1–7 post-exposure followed by steady recovery to baseline by Day 14 (Table 6).

#### 3.3.4. Necropsy Findings

Gross and histopathological assessments revealed no exposure-related abnormalities in vital organs (lungs, liver, kidneys), confirming TFSF’s reversible toxicity profile.

### 3.4. Summary

The acute inhalation LC_50_ for TFSF exceeds 22,609.9 ppm, aligning with Category 5 toxicity (OECD Test Guideline 403 [22])—a classification reserved for substances with >20,000 ppm LC_50_. Combined with rapid clinical recovery and absence of chronic pathology, these findings validate TFSF’s suitability as a low-risk SF_6_ alternative in high-voltage insulation systems.

## 4. Conclusions

This research establishes an eco-conscious synthesis protocol for TFSF through catalytic halogen exchange, employing CF_3_SO_2_Cl and KF under crown ether mediation. Optimized at a 3:1 KF ratio and controlled low temperatures, the method delivers 65% isolated yield with 97.9% purity—metrics retained during pressurized reactor scale-up trials demonstrating 58% conversion and sustained 94% selectivity. Notably, standardized inhalation toxicity assessments revealed dose-responsive safety: Sprague-Dawley rats exposed to 20,000 ppm TFSF for 4 h exhibited transient neurological effects with 66% survival, while Probit analysis determined an LC_50_ exceeding 22,600 ppm. This dual validation of scalable synthesis and a favorable toxicological profile positions TFSF as a viable SF_6_ substitute, offering the power sector an actionable pathway to reduce fluorinated greenhouse emissions.

## Figures and Tables

**Table 1:** Influence of feedstock ratio on TFSF yield and purity.

Ratio (KF:CF_3_SO_2_Cl)	Reaction Temperature (°C)	Yield (%)	Purity (%)
1:1	0	48	90.0
2:1	0	53	91.2
3:1	0	65	97.9
4:1	0	63	98.2
5:1	0	65	96.7

**Table 2:** Effect of reaction temperature on TFSF synthesis.

Ratio (KF:CF_3_SO_2_Cl)	Reaction Temperature (°C)	Yield (%)	Purity (%)
3:1	−10	21	96.2
3:1	0	65	97.9
3:1	20	54	90.6
3:1	40	48	78.2
3:1	60	45	43.5

**Table 3:** Batch scale-up experimental results.

Batch No.	CF_3_SO_2_Cl (kg)	Anhydrous KF (kg)	Crude Product (kg, 60% Purity)	Final Product (kg, >99% Purity)
1	2	0.69	1.52	2.77
2	2	0.69	1.58	
3	2.5	0.86	1.89	
4	2.5	0.86	1.94	

**Table 4:** Experimental animal information.

Parameter	Value/Range
Species/Strain	Sprague-Dawley (SD) rat
Health Status	SPF
Number and Gender	3 males, 3 females
Body Weight Range (at Exposure Start)	Females: 245–267 g; Males: 357–388 g
Supplier	Specified Biotechnology Company (Beijing, China)

**Table 5:** Summary of mortality data.

Actual Exposure Concentration (ppm)	Sex	Mortality (Number/Total)
22,609.9	Male	1/3
22,609.9	Female	1/3

**Table 6:** Body weight change summary (g).

Actual Exposure (ppm)	Sex	Δ Weight Day 7–Day 0 (g)	Δ Weight Day 14–Day 7 (g)
22,609.9	Female	−3.5 ± 3.5	11.0 ± 2.8
22,609.9	Male	7.5 ± 7.8	29.5 ± 4.9

## Data Availability

The original contributions presented in this study are included in the article. Further inquiries can be directed to the corresponding author(s).
